# Elevated growth differentiation factor-15 in sepsis: clinical associations and immune cell context

**DOI:** 10.3389/fcell.2026.1789747

**Published:** 2026-03-16

**Authors:** Wenzhe Li, Yuqian Li, Yixi Wang, Ziwei Wu, Jian Cui, Jialing Wang, Xiangyou Yu

**Affiliations:** 1 Department of Critical Care Medicine, the First Affiliated Hospital of Xinjiang Medical University, Urumqi, Xinjiang, China; 2 Department of Anesthesiology, the First Affiliated Hospital of Xinjiang Medical University, Urumqi, Xinjiang, China; 3 Department of Minimally Invasive Spine and Precision Orthopedics, the First Affiliated Hospital of Xinjiang Medical University, Urumqi, Xinjiang, China

**Keywords:** biomarkers, disease severity, growth differentiation factor-15 (GDF-15), Immunestress, sepsis, single-cell RNA sequencing

## Abstract

**Background:**

Early clinical assessment in sepsis remains challenging because of marked heterogeneity in host responses and the limited biological resolution of existing severity scores and conventional inflammatory biomarkers in capturing integrated cellular stress responses. Growth differentiation factor-15 (GDF-15) is a stress-responsive cytokine that is elevated in critical illness; however, its clinical associations and immune cell-specific context in sepsis remain incompletely characterized.

**Methods:**

In this prospective observational exploratory study, serum GDF-15 concentrations were measured by enzyme-linked immunosorbent assay in a small cohort of adult patients with sepsis (n = 19) and healthy controls (n = 23). Associations between circulating GDF-15 levels, disease severity scores, markers of organ dysfunction, and inflammatory biomarkers were assessed using non-parametric correlation analyses. To provide biological context, publicly available single-cell RNA sequencing data (GSE217906) were analyzed to characterize immune cell-specific patterns of GDF15 expression during acute sepsis.

**Results:**

Circulating GDF-15 concentrations were markedly elevated in patients with sepsis compared with healthy controls (median 1390 vs. 1050 pg/mL). Among patients with sepsis, higher GDF-15 levels were consistently observed in those with greater disease severity, and increased levels of inflammatory biomarkers. Analysis of independent single-cell transcriptomic data demonstrated increased GDF15 expression in immune cells during acute sepsis, with relative enrichment observed primarily in plasma cells and monocytes. Exploratory receiver operating characteristic analyses suggested that circulating GDF-15 exhibited descriptive discrimination patterns for 28-day mortality that were directionally similar to those of established severity scores and conventional inflammatory biomarkers.

**Conclusion:**

Circulating GDF-15 levels are elevated in sepsis and are associated with disease severity, organ dysfunction, and inflammatory activity. Immune cell-specific enrichment of GDF15 expression provides biological context for its elevation during acute sepsis. Together, these findings suggest that GDF-15 may reflect, at least in part, systemic biological stress in critical illness, warranting further investigation in larger, well-characterized cohorts.

## 1 Introduction

Sepsis is a life-threatening condition characterized by organ dysfunction resulting from a dysregulated host response to infection and remains a leading cause of mortality in intensive care units worldwide (Rudd et al., 2020). Despite advances in supportive care and management strategies, sepsis continues to be associated with high morbidity, prolonged intensive care unit stays, and substantial short-term mortality (Singer et al., 2016). A persistent clinical challenge lies in the marked heterogeneity of host responses during acute illness, which complicates early identification and risk stratification of patients who develop severe organ dysfunction (Russell, 2019). Current approaches rely predominantly on clinical severity scores such as Sequential Organ Failure Assessment (SOFA) and the Acute Physiology and Chronic Health Evaluation II (APACHE II) (Cecconi et al., 2014; Evans et al., 2021). While indispensable for standardized assessment, these instruments primarily capture downstream physiological derangements rather than the underlying biological stress responses driving disease progression (Tekin et al., 2024).

Conventional inflammatory biomarkers, including C-reactive protein (CRP) and procalcitonin (PCT), are widely used to support infection diagnosis and monitor inflammatory burden (Peng et al., 2026). Although clinically useful, these markers are largely confined to inflammatory activity and lack specificity for immune dysregulation. Importantly, they do not adequately capture broader cellular stress responses, metabolic perturbations, or immune exhaustion, all of which are increasingly recognized as central features of sepsis pathophysiology, particularly during the transition from early hyperinflammation to subsequent immunosuppressive states (Chadda and Puthucheary, 2024). Accordingly, sepsis is increasingly conceptualized as a heterogeneous immunological syndrome rather than a uniform inflammatory condition (Gao et al., 2025). Biomarkers that reflect integrated cellular stress responses may therefore provide complementary biological information beyond conventional inflammatory markers and severity scores. Growth differentiation factor-15 (GDF-15) is a stress-responsive cytokine belonging to the transforming growth factor β superfamily and is induced by a wide range of cellular stressors, including inflammation, hypoxia, oxidative stress, and mitochondrial dysfunction (Desmedt et al., 2019). Circulating GDF-15 levels have been reported to increase across diverse clinical settings characterized by systemic stress, such as cardiovascular disease, metabolic disorders, malignancy, and critical illness (Wischhusen et al., 2020). Notably, GDF-15 is increasingly regarded as a marker of global cellular stress rather than a classical pro-inflammatory mediator (Silva-Bermudez et al., 2024; Clark et al., 2013). However, the lack of disease specificity of GDF-15 has raised concerns regarding its interpretability in complex syndromes such as sepsis (Benarroch, 2025). In the context of sepsis, several studies have reported elevated circulating GDF-15 concentrations and associations with disease severity and adverse outcomes (Desmedt et al., 2019; Kuang et al., 2025). However, most existing investigations have focused primarily on serum measurements and clinical correlations, with limited attention to the immune cellular context in which GDF-15 is expressed (Lin et al., 2026). Consequently, the biological interpretation of elevated circulating GDF-15 levels in sepsis remains incomplete (Hüllwegen et al., 2025).

Recent advances in single-cell RNA sequencing have enabled high-resolution characterization of immune heterogeneity in critical illness, revealing profound alterations in both innate and adaptive immune compartments during sepsis (Chambers et al., 2019; Stanski and Wong, 2020). These approaches provide an opportunity to situate circulating biomarkers within a cellular and transcriptional framework, thereby enhancing biological interpretability. Integrating clinical biomarker data with immune cell-specific transcriptomic information may therefore yield valuable insight into stress-related processes underlying sepsis-associated immune dysregulation. In this exploratory study, we combined prospective clinical biomarker profiling with analysis of publicly available single-cell transcriptomic data to examine the clinical associations and immune cell context of GDF-15 in adult patients with sepsis. Specifically, we aimed to (i) evaluate relationships between circulating GDF-15 levels and established severity scores, organ dysfunction markers, and inflammatory biomarkers; and (ii) characterize immune cell-specific patterns of GDF15 expression during acute sepsis. By integrating clinical and cellular data, this study seeks to provide biological context for GDF-15 elevation in sepsis, rather than to establish diagnostic or prognostic utility.

## 2 Materials and methods

### 2.1 Study design and participants

This study was designed as a prospective, observational, exploratory investigation conducted in the intensive care unit (ICU) of the First Affiliated Hospital of Xinjiang Medical University between September 2024 and August 2025. Adult patients with sepsis were consecutively enrolled and compared with healthy controls recruited during routine health examinations. The study protocol was approved by the Ethics Committee of the First Affiliated Hospital of Xinjiang Medical University (Approval No. K202404-45). Written informed consent was obtained from all participants or their legally authorized representatives prior to enrollment.

Sepsis was defined according to the Sepsis-3 criteria (Singer et al., 2016) as life-threatening organ dysfunction caused by a dysregulated host response to infection. Inclusion criteria for the sepsis group were: (Rudd et al., 2020): age ≥18 years; (Singer et al., 2016); fulfillment of Sepsis-3 diagnostic criteria; and (Russell, 2019) admission to the ICU. Exclusion criteria included active malignancy, age <18 years, ICU stay shorter than 24 h, or absence of sepsis. Healthy controls were adult volunteers without acute infection, chronic inflammatory disease, or known immune disorders (Supplementary Figure S1).

### 2.2 Clinical data collection

Baseline demographic and clinical data were collected at the time of ICU admission, including age, sex, body weight, vital signs, and routine laboratory parameters. Disease severity was assessed using the SOFA and APACHE II scores. Laboratory variables included white blood cell count (WBC), hemoglobin, platelet count, serum creatinine, blood urea nitrogen (BUN), electrolytes, CRP, PCT, and interleukin-6 (IL-6). Use of vasopressor therapy during the ICU stay was recorded as a marker of circulatory support. All clinical data were extracted from electronic medical records by trained investigators who were blinded to biomarker measurements.

### 2.3 Blood sample collection and biomarker measurements

Peripheral venous blood samples were obtained from all participants, within 24 h of ICU admission for patients with sepsis and during routine blood sampling for healthy controls. Samples were centrifuged at 3000 *g* for 10 min to isolate serum, which was aliquoted and stored at −80 °C until analysis. Serum concentrations of GDF-15, interleukin-6 (IL-6), CRP, and procalcitonin (PCT) were measured using commercially available enzyme-linked immunosorbent assay (ELISA) kits according to the manufacturers’ instructions: GDF-15 (Human GDF-15 ELISA Kit, Cat. No. E-EL-H0080; Elabscience, Wuhan, China), PCT (Human procalcitonin ELISA Kit, Cat. No. E-EL-H1492; Elabscience, Wuhan, China), IL-6 (Human interleukin-6 ELISA Kit, Cat. No. KE00385; Proteintech, Wuhan, China), and CRP (Human C-reactive protein ELISA Kit, Cat. No. KE00004; Proteintech, Wuhan, China). All measurements were performed in duplicate by trained laboratory personnel blinded to clinical data and outcomes. No imputation of missing biomarker values was performed.

### 2.4 Single-cell RNA sequencing data acquisition

To provide biological and cellular context for circulating GDF-15 levels, publicly available single-cell RNA sequencing (scRNA-seq) data were obtained from the Gene Expression Omnibus (GEO) database (accession number GSE217906) (Sun et al., 2025). This dataset comprises peripheral blood mononuclear cell transcriptomes from healthy controls, patients with acute sepsis, and patients with post-sepsis persistent inflammation, immunosuppression, and catabolism syndrome (PICS). For the present analysis, only samples from healthy controls and patients with acute sepsis were included. The scRNA-seq analysis was conducted independently of the clinical cohort and was intended to provide contextual biological insight rather than patient-level validation of circulating biomarker findings.

### 2.5 scRNA-seq data processing and quality control

scRNA-seq data were processed using the Seurat package (version 5.3) (Hao et al., 2024). Cells expressing fewer than 200 genes or more than 8,000 genes were excluded to remove low-quality cells and potential doublets. Cells with mitochondrial gene expression exceeding 10% or hemoglobin gene expression exceeding 2.5% were removed to minimize technical artifacts and contamination from damaged or erythroid-derived cells. These quality control thresholds were selected based on established practices in prior sepsis scRNA-seq studies and visual inspection of quality metrics to balance data quality and cell retention. After quality control, a total of 66,731 high-quality cells were retained for downstream analyses. Gene expression values were normalized using the NormalizeData function with a scaling factor of 10,000, followed by log transformation. Highly variable genes (n = 2,000) were identified using the FindVariableFeatures function. Data were scaled using ScaleData, and principal component analysis (PCA) was performed. The first 40 principal components were selected for downstream analyses based on variance explained and inspection of elbow plots. Graph-based clustering was performed using the FindNeighbors and FindClusters functions.

Comparisons of GDF15 expression across conditions and immune cell types were performed for descriptive and exploratory purposes to facilitate qualitative interpretation of cell type–associated expression patterns. Gene expression values were reported as log-normalized counts. For functional interpretation, Gene Ontology (GO) biological process enrichment analysis was performed on immune cell subsets expressing GDF15 using ClueGO. Functional enrichment results were presented to support descriptive interpretation of stress-related biological processes.

### 2.6 Statistical analysis

Clinical and biomarker data were analyzed using R software (version 4.4.3). Continuous variables were presented as medians with interquartile ranges (IQRs), and categorical variables as counts and percentages. Between-group comparisons were performed using the Mann–Whitney U test for continuous variables and the Chi-square test for categorical variables. To account for the significant age difference between the sepsis and control groups, an analysis of covariance (ANCOVA) was additionally performed. GDF-15 values were log-transformed to satisfy normality assumptions, and the model included group (sepsis vs. control) as a fixed factor and age as a covariate. Associations between circulating GDF-15 levels, disease severity scores, markers of organ dysfunction, and inflammatory biomarkers were assessed using Spearman’s rank correlation coefficients (two-sided). This non-parametric approach was selected due to the small sample size and non-normal data distributions. Scatter plots were provided for visualization only and include fitted linear regression trends with 95% confidence intervals. Exploratory receiver operating characteristic (ROC) analyses were conducted to visualize the relative discrimination patterns of circulating GDF-15, inflammatory biomarkers, and clinical severity scores for 28-day mortality among patients with sepsis. Areas under the curve (AUCs) with 95% confidence intervals were calculated. No multivariable modeling, internal validation, or cutoff optimization was performed. All ROC analyses were explicitly considered descriptive and hypothesis-generating. A two-sided p-value <0.05 was considered statistically significant.

## 3 Results

### 3.1 Patient characteristics and clinical severity

A total of 42 participants were included in this study, comprising 19 patients with sepsis and 23 healthy controls. Baseline demographic and clinical characteristics are summarized in Table 1. Patients with sepsis were older than healthy controls (61.3 vs. 44.5, *p* = 0.001; Supplementary Table S1) and exhibited marked physiological abnormalities at ICU admission, including higher heart rate, respiratory rate, and body temperature, as well as lower diastolic blood pressure and peripheral oxygen saturation. Disease severity in the sepsis group was substantial, as reflected by elevated SOFA score (median 12.0 [IQR 6.50]) and APACHE II score (median 28.0 [IQR 10.5]) at enrollment. Laboratory findings indicated systemic inflammation and multiorgan involvement in patients with sepsis, with higher white blood cell counts, blood urea nitrogen, and serum creatinine levels, as well as lower hemoglobin and platelet counts. More than half of the patients with sepsis (52.6%) required vasopressor support during their ICU stay, consistent with significant circulatory dysfunction.

**TABLE 1:** Characteristics of the study population in the first affiliated hospital of xinjiang medical university.

Variables	All patients (n = 42)	Control group (n = 23)	Sepsis group (n = 19)	*p*-value
Age (years, M [IQR])	50.1 [21.7]	44.5 [15.8]	61.3 [17.9]	0.001
Gender (Male, n (%))	17 (40.5)	6 (26.1)	11 (57.9)	0.076
Weight (kg, M [IQR])	74.9 [14.7]	74.9 [17.4]	76.2 [13.9]	0.849
SOFA (M [IQR])	0 [11.0]	0 [0]	12.0 [6.50]	<0.001
APACHE II(M [IQR])	0 [27.0]	0 [0]	28.0 [10.5]	<0.001
Vital Signs (M [IQR])				
Heart rate (beats/min)	72.5 [22.3]	60.0 [17.0]	88.0 [43.0]	<0.001
Systolic blood pressure (mmHg)	105 [9.57]	106 [8.50]	103 [9.59]	0.216
Diastolic blood pressure (mmHg)	53.5 [16.8]	58.0 [7.50]	41.0 [11.0]	<0.001
Respiratory rate (breaths/min)	13.5 [8.00]	10.0 [3.00]	21.0 [12.0]	<0.001
Temperature (°C)	36.8 [0.550]	36.7 [0.250]	37.2 [0.355]	<0.001
SpO_2_ (%)	100 [2.75]	100 [0]	97.0 [4.00]	0.002
Laboratory Parameters (M [IQR])				
White blood cell (×10^9^/L)	11.2 [7.68]	10.0 [4.40]	17.2 [10.2]	<0.001
Hematocrit (%)	37.8 [8.78]	39.8 [3.60]	31.4 [10.2]	<0.001
Hemoglobin (g/dL)	12.5 [2.35]	13.3 [1.20]	10.8 [3.20]	<0.001
Platelets (×10^9^/L)	227 [115]	244 [114]	117 [160]	<0.001
Blood urea nitrogen (mg/dL)	12.5 [17.3]	10.0 [4.00]	27.0 [42.5]	<0.001
Serum creatinine (μmol/L)	70.8 [68.6]	53.1 [8.85]	142 [159]	<0.001
Sodium (mmol/L)	136 [7.00]	137 [4.00]	130 [5.50]	<0.001
Potassium (mmol/L)	3.80 [0.55]	3.80 [0.30]	4.00 [0.95]	<0.001
Blood Glucose (mmol/L)	5.92 [2.18]	5.89 [2.00]	6.22 [2.22]	0.765
Procalcitonin (ng/mL)	0.08 [0.64]	0.01 [0.01]	0.93 [2.24]	0.008
CRP (mg/dL)	1.29 [17.7]	0.490 [0.520]	19.3 [17.8]	<0.001
GDF15 (pg/mL)	1130 [306]	1050 [93.2]	1390 [143]	<0.001
IL-6 (pg/mL)	523 [248]	480 [63.0]	732 [36.6]	<0.001
Use of Vasopressors (n (%))	10 (23.8%)	0 (0%)	10 (52.6%)	<0.001
28d mortality (n (%))	8 (19.0%)	0 (0%)	8 (42.1%)	<0.001

### 3.2 Circulating GDF-15 levels in patients with sepsis and healthy controls

**FIGURE 1:**
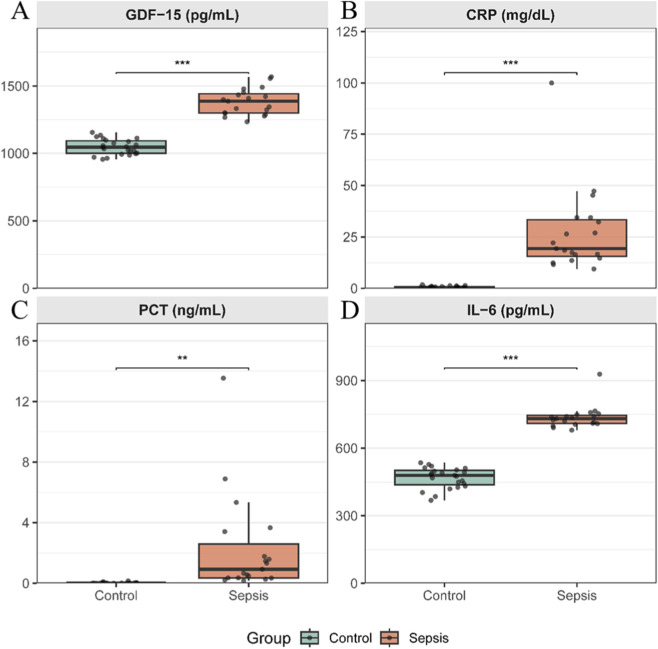
Circulating levels of inflammatory biomarkers in patients with sepsis and healthy controls Box-and-whisker plots comparing serum levels of **(A)** growth differentiation factor-15 (GDF-15), **(B)** C-reactive protein (CRP), **(C)** procalcitonin (PCT), and **(D)** interleukin-6 (IL-6) between healthy controls (n = 23) and patients with sepsis (n = 19). Boxes represent the interquartile range (IQR) with the median indicated by a horizontal line; whiskers denote 1.5 × IQR, and individual data points are overlaid. Between-group comparisons were performed using the two-sided Mann–Whitney U test. **: p < 0.01, ***: p < 0.001.

Serum concentrations of growth differentiation factor-15 (GDF-15) were markedly elevated in patients with sepsis compared with healthy controls (median 1390 [IQR 143] pg/mL vs. 1050 [93.2] pg/mL, *p* < 0.001; Figure 1A). Substantial inter-individual variability in circulating GDF-15 levels was observed within the sepsis group. Consistent with the inflammatory state of sepsis, circulating levels of CRP (median 19.30 [IQR 17.80] mg/dL vs. 0.49 [IQR 0.52] mg/dL), IL-6 (median 732 [IQR 36.6] pg/mL vs. 480 [IQR 63.0] pg/mL), and PCT (median 10.93 [IQR 2.24] ng/mL vs. 0.01 [IQR 0.01] ng/mL) were also higher in patients with sepsis than in healthy controls, all *p* < 0.01 (Figures 1B–D). After adjusting for age, the difference between groups remained statistically significant (F [1,39] = 10.2, *p* = 0.003), indicating that the elevation of GDF-15 in sepsis is independent of age (Supplementary Table S2).

### 3.3 Associations between circulating GDF-15 levels and disease severity

**FIGURE 2:**
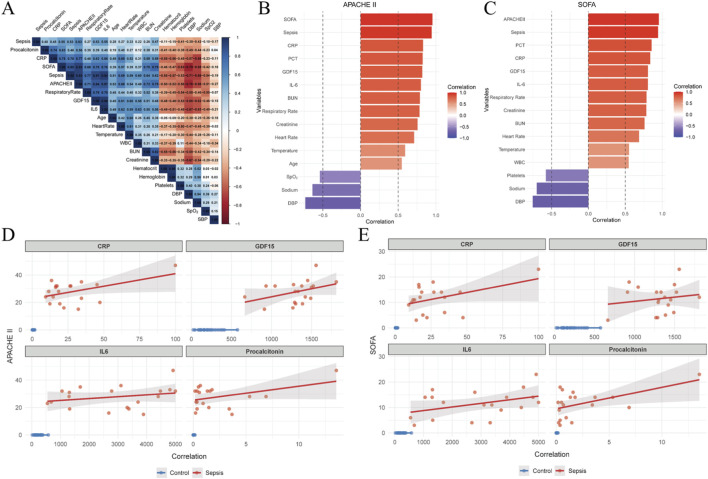
Associations between circulating GDF-15 levels, disease severity, and inflammatory biomarkers. **(A)** Heatmap summarizing Spearman correlation coefficients between circulating GDF-15 levels, disease severity scores, and sepsis status. **(B,C)** Scatter plots illustrating associations between circulating GDF-15 concentrations and APACHE II score **(B)** and SOFA score **(C)** in patients with sepsis. **(D,E)** Scatter plots illustrating associations between circulating inflammatory biomarkers and APACHE II score **(D)** and SOFA score **(E)**. Correlation analyses were performed using Spearman’s rank correlation. Scatter plots are provided for descriptive visualization; solid lines indicate fitted linear regression trends with 95% confidence intervals.

A correlation heatmap summarizing relationships between circulating GDF-15 levels, disease severity scores, and sepsis status is shown in Figure 2A and is provided for descriptive visualization. Associations between circulating GDF-15 concentrations and clinical severity among patients with sepsis were assessed using Spearman’s rank correlation analyses, as predefined in the Methods. Circulating GDF-15 levels demonstrated strong positive monotonic associations with established severity scores, including the SOFA score (Spearman’s ρ = 0.75, *p* < 0.001) and the APACHE II score (Spearman’s ρ = 0.84, p < 0.001) (Figures 2B,C). Higher GDF-15 concentrations were consistently observed in patients with greater overall disease severity.

### 3.4 Associations between circulating GDF-15 levels and inflammatory biomarkers

Associations between circulating GDF-15 levels and conventional inflammatory biomarkers were further examined in patients with sepsis. Serum GDF-15 concentrations were positively correlated with IL-6 (Spearman’s ρ = 0.86, *p* < 0.001) and CRP (Spearman’s ρ = 0.63, *p* < 0.001) (Figures 2D,E). These associations indicate that elevated circulating GDF-15 levels tended to co-occur with higher inflammatory biomarker levels in this cohort. All correlation analyses were exploratory in nature.

### 3.5 Immune cell-specific expression patterns of GDF15 in acute sepsis

**FIGURE 3:**
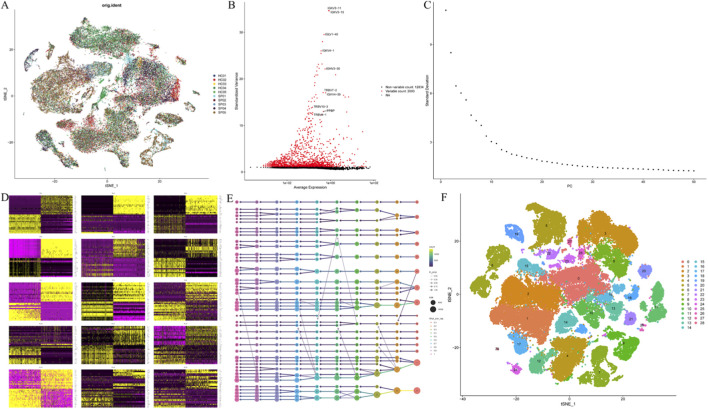
Quality control, dimensionality reduction, and clustering of single-cell RNA sequencing data. **(A)** Integration of single-cell transcriptomic data from healthy controls and patients with acute sepsis following batch correction, showing overall alignment of datasets. **(B)** Identification of the top 2,000 highly variable genes used for downstream analyses. **(C,D)** Principal component analysis (PCA) of the integrated dataset; the first 40 principal components were selected based on variance explained and elbow plot inspection. **(E)** Graph-based clustering performed after evaluation of multiple resolutions (0.1–1.0), with a final resolution of 0.4 selected for downstream analyses. **(F)** Two-dimensional t-distributed stochastic neighbor embedding (t-SNE) visualization illustrating identified cell clusters.

To provide cellular context for the observed elevation of circulating GDF-15 levels, publicly available single-cell RNA sequencing data were analyzed independently of the clinical cohort. After quality control filtering (Supplementary Figures S2-S3), multiple clustering resolutions ranging from 0.1 to 1.0 were evaluated, and a final resolution of 0.4 was selected based on cluster stability and biological interpretability (Figure 3). Dimensionality reduction for visualization was conducted using t-distributed stochastic neighbor embedding (t-SNE). Cell clusters were annotated into major immune cell populations using established canonical marker genes, including LYZ and CD14 for monocytes, MS4A1 for B cells, MZB1 for plasma cells, GNLY for natural killer cells, IL7R and MAL for CD4^+^ T cells, GZMA and CD8A for CD8^+^ T cells, CSF3R for neutrophil cells, TRDV2 for γδ T cells, and PPBP for platelets. A total of nine major immune cell populations were identified (Supplementary Figure S4). A total of 66,731 high-quality peripheral blood immune cells were retained and classified into nine major immune cell populations (Figure 3; Supplementary Figure S4).

**FIGURE 4:**
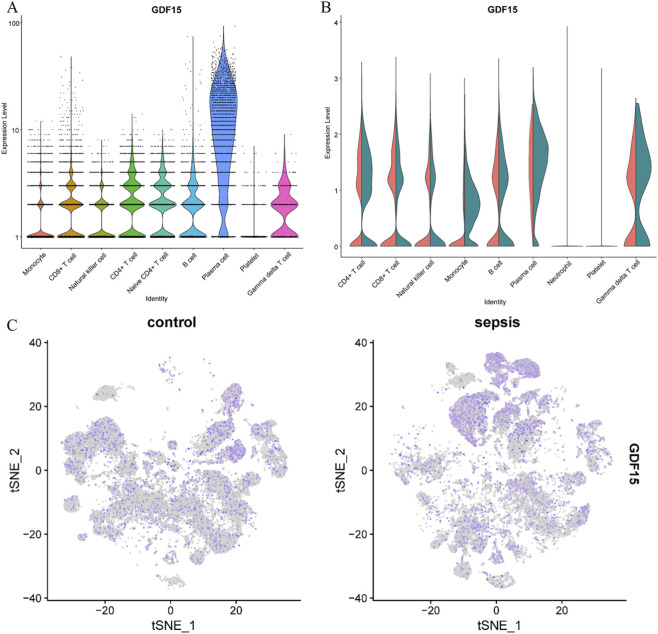
Distribution of GDF15 expression across immune cell populations. **(A)** Violin plots showing log-normalized GDF15 expression across major immune cell populations. **(B)** Comparison of GDF15 expression between healthy controls and patients with acute sepsis within each immune cell population. **(C)** GDF15 expression in selected immune cell populations in patients with sepsis. GDF15 expression patterns across immune cell populations are shown for descriptive visualization.

Comparative visualization revealed increased GDF15 transcript expression in immune cells from patients with acute sepsis compared with healthy controls (Figure 4). Among immune cell populations, plasma cells exhibited the most pronounced relative enrichment of GDF15 expression, followed by monocytes. More modest increases were observed in CD4^+^ T cell subsets. Overall, GDF15 expression displayed heterogeneous distribution across immune cell populations, indicating cell type-specific transcriptional responses during acute sepsis. These comparisons were intended to provide qualitative and descriptive insight into immune cell-specific expression patterns. Functional enrichment analysis of GDF15-expressing immune cell subsets suggested enrichment of biological processes related to cellular stress responses and immune activation (Supplementary Figure S5).

### 3.6 Exploratory ROC analysis for 28-day mortality

Exploratory ROC analyses were performed to visualize the relative discrimination patterns of circulating GDF-15 levels, inflammatory biomarkers, and clinical severity scores for 28-day mortality among patients with sepsis (Supplementary Figure S6). In an exploratory logistic regression analysis adjusting for age, GDF-15 was not independently associated with 28-day mortality (OR per 100 pg/mL increase = 1.08, 95% CI 0.91–1.28, *p* = 0.38; Supplementary Table S3). These analyses were conducted for descriptive visualization only and were not intended to assess prognostic performance, given the limited sample size and number of outcome events. Accordingly, GDF-15 should not be considered a prognostic biomarker based on the present data.

## 4 Discussion

In this exploratory study, we examined the clinical associations and immune cell-specific context of GDF-15 in adult patients with sepsis by integrating prospective serum biomarker measurements with publicly available single-cell transcriptomic data. Several observations emerged from this analysis. First, circulating GDF-15 concentrations were elevated in patients with sepsis compared with healthy controls. Second, higher GDF-15 levels were associated with greater disease severity, markers of organ dysfunction, and increased levels of inflammatory biomarkers. Third, independent single-cell RNA sequencing analysis revealed immune cell-specific enrichment of GDF15 transcript expression during acute sepsis, most prominently in plasma cells and monocytes. Collectively, these findings suggest that elevated GDF-15 may reflect the burden of systemic biological stress accompanying severe sepsis, rather than representing a standalone diagnostic or prognostic marker (Buendgens et al., 2017).

Sepsis is increasingly recognized as a heterogeneous syndrome characterized by dynamic and patient-specific host responses. Established clinical severity scores, such as SOFA and APACHE II, remain central to risk stratification and clinical decision-making but primarily capture downstream physiological derangements and established organ dysfunction (Davoulos et al., 2025; Guo et al., 2025). Similarly, conventional inflammatory biomarkers, including CRP and PCT, provide information on systemic inflammatory activity but offer limited insight into broader cellular stress responses (Yang et al., 2024). Within this context, the observed associations between circulating GDF-15 levels and established severity scores in the present study indicate that GDF-15 increases in parallel with overall disease severity, rather than reflecting an isolated inflammatory pathway (Lin et al., 2026).

The strong monotonic correlations observed between GDF-15 concentrations and severity indices should be interpreted cautiously. Given the exploratory design and limited sample size, these findings do not imply independence from, or superiority over, existing clinical scores (Ito et al., 2025). Moreover, components of severity scoring systems overlap with markers of organ dysfunction that were also associated with GDF-15 levels, which may partially account for the observed relationships. Rather than supporting a causal or predictive role, the results are most consistent with the interpretation that circulating GDF-15 tracks integrated physiological stress across multiple organ systems during severe sepsis (Abulizi et al., 2017).

Associations between circulating GDF-15 levels and markers of sepsis further support this interpretation. Severe systemic inflammatory response syndrome is a frequent complication of sepsis and a major contributor to disease severity and adverse outcomes (Fan et al., 2026). In this study, higher GDF-15 concentrations were associated with elevated serum CRP and IL-6 levels. Unlike classical pro-inflammatory cytokines, GDF-15 is induced by a broad range of stressors, encompassing inflammatory, hypoxic, oxidative, and metabolic signals (Peksöz et al., 2022). Consequently, elevated GDF-15 levels may reflect the cumulative burden of inflammatory and non-inflammatory stress rather than inflammatory activity alone (Clark et al., 2013). This broader stress-responsive profile may explain why GDF-15 aligns with, yet is not redundant to, conventional inflammatory biomarkers in sepsis (Clark et al., 2013; Buendgens et al., 2017). Notably, age-adjusted analysis confirmed that the elevation of GDF-15 in sepsis was independent of age and not merely an artifact of the age difference between groups. The association with mortality, however, was weak and non-significant after age adjustment, consistent with the limited statistical power of this exploratory cohort. These findings support the robustness of the primary observation that GDF-15 is elevated in sepsis independent of age, while underscoring the need for larger studies to evaluate prognostic utility.

To provide cellular context for these clinical associations, we analyzed publicly available single-cell RNA sequencing data derived from an independent cohort (Sun et al., 2025). This analysis demonstrated increased GDF15 transcript expression in immune cells from patients with acute sepsis, with enrichment observed primarily in plasma cells and monocytes. These immune subsets are known to experience substantial metabolic and inflammatory stress during sepsis, making them plausible contributors to stress-responsive transcriptional programs (Stanski and Wong, 2020). This cell-type specificity links the circulating biomarker to distinct immune subsets under stress, supporting the interpretation of GDF-15 as a marker of integrated cellular stress. While the data do not identify the cellular sources of circulating GDF-15, they establish a cell-specific framework that generates testable hypotheses for future mechanistic studies. Importantly, the single-cell findings are intended to offer biological plausibility and contextual insight rather than direct evidence of the cellular sources of circulating GDF-15 (Melero et al., 2025). Exploratory receiver operating characteristic analyses suggested that circulating GDF-15 exhibited descriptive discrimination patterns for 28-day mortality that were directionally similar to those of established severity scores and inflammatory biomarkers (Karakike et al., 2019). These analyses were performed solely for visualization purposes and were not designed to assess prognostic performance. Accordingly, GDF-15 should not be considered a prognostic biomarker based on the present data.

Several limitations of this study warrant consideration. First, the single-center design and modest sample size limit generalizability and statistical robustness. Second, the absence of formal age matching between patients with sepsis and healthy controls may have influenced observed differences in circulating GDF-15 levels, given known age-related variation in this biomarker. Third, biomarker measurements were obtained at a single time point, precluding assessment of temporal dynamics during disease progression. Finally, the use of independent single-cell transcriptomic data provides valuable contextual insight but does not allow direct linkage between circulating and cellular findings.

## 5 Conclusion

In conclusion, this exploratory study demonstrates that circulating GDF-15 levels are elevated in sepsis and are associated with disease severity, organ dysfunction, and immune cell-specific stress-related transcriptional patterns. These findings support the interpretation of GDF-15 as a marker reflecting systemic biological stress in critical illness. Larger, multicenter studies incorporating longitudinal sampling and integrated clinical and cellular analyses will be required to clarify the potential role of GDF-15 as a complementary biomarker in sepsis and to determine its relevance beyond established clinical indices.

## Data Availability

The datasets presented in this study can be found in online repositories. The names of the repository/repositories and accession number(s) can be found in the article/Supplementary Material.
