# Supplementary material for: Elevated growth differentiation factor-15 in sepsis: clinical associations and immune cell context
Source: Front Cell Dev Biol. 2026 Mar 16;14:1789747. doi: 10.3389/fcell.2026.1789747 (PMC13033658; doi:10.3389/fcell.2026.1789747)
Supplement: Supplementary file 1 [file Supplementaryfile1.docx]

**Supplementary table files**

**Supplementary Table S1** Age and GDF‑15 levels in the study population

**Supplementary Table S2** ANCOVA for log(GDF‑15) with group and age

**Supplementary Table S3** Logistic regression for 28‑day mortality

**Supplementary Figure Legends**

**Supplementary Figure S1.** Schematic overview of patient selection and study procedures.

**Supplementary Figure S2.** Quality control metrics of single-cell RNA sequencing data.

**Supplementary Figure S3.** Cell filtering strategy for single-cell RNA sequencing analysis.

**Supplementary Figure S4.** Cell-type identification and annotation of peripheral blood immune cells.

**Supplementary Figure S5.** Functional enrichment analysis of GDF15-expressing immune cell subsets.

**Supplementary Figure S6.** Exploratory receiver operating characteristic (ROC) analysis for 28-day mortality.

**Supplementary Table S1.** Age and GDF‑15 levels in the study population

| Variable | Control (n = 23) | Sepsis (n = 19) | *p*‑value |
| --- | --- | --- | --- |
| Age, years, median [IQR] | 44.5 [15.8] | 61.3 [17.9] | < 0.001 |
| GDF‑15, pg/mL, median [IQR] | 1050 [93.2] | 1390 [143] | < 0.001 |
| log(GDF‑15), mean (SD) | 6.96 (0.09) | 7.24 (0.10) | – |

Supplementary Table S1 presents the age and GDF‑15 distributions for the two groups. Patients with sepsis were significantly older than healthy controls (median 61.3 vs. 44.5 years, *p* < 0.001). Raw GDF‑15 levels were markedly higher in the sepsis group (median 1390 vs. 1050 pg/mL, *p* < 0.001).

**Supplementary Table S2.** ANCOVA for log(GDF‑15) with group and age

| Source | Sum of Squares | df | Mean Square | F | *p*‑value | Partial η² |
| --- | --- | --- | --- | --- | --- | --- |
| Group | 0.412 | 1 | 0.412 | 10.23 | 0.003 | 0.21 |
| Age | 0.089 | 1 | 0.089 | 2.21 | 0.145 | 0.05 |
| Residual | 1.572 | 39 | 0.040 |  |  |  |

Model R² = 0.24, adjusted R² = 0.21.

To adjust for age, an analysis of covariance (ANCOVA) was performed with log‑transformed GDF‑15 as the dependent variable. Log transformation was applied to satisfy the normality and homoscedasticity assumptions of ANCOVA (Shapiro–Wilk test on residuals: *p* = 0.28). The model included group (sepsis vs. control) as a fixed factor and age as a covariate. After adjusting for age using analysis of covariance (ANCOVA) with log‑transformed GDF‑15, the difference between the sepsis and control groups remained statistically significant (F(1, 39) = 10.23, *p* = 0.003, partial η² = 0.21), indicating that the elevation of GDF‑15 in sepsis is independent of age. Age itself was not a significant predictor in the model (*p* = 0.145). The full ANCOVA results are presented in Supplementary Table S2.

**Supplementary Table S3.** Logistic regression for 28‑day mortality

| Variable | OR (95% CI) | *p*‑value |
| --- | --- | --- |
| GDF‑15 (per 100 pg/mL) | 1.08 (0.91–1.28) | 0.38 |
| Age (per year) | 1.04 (0.98–1.11) | 0.21 |
| Model statistics |  |  |
| –2 Log likelihood | 21.34 |  |
| Nagelkerke R² | 0.11 |  |
| Hosmer–Lemeshow test | χ²(8) = 5.67, *p* = 0.68 |  |

In the unadjusted logistic regression, GDF‑15 showed a trend toward association with mortality (OR per 100 pg/mL increase = 1.15, 95% CI 0.98–1.35, *p* = 0.09). After adjusting for age, the effect was attenuated and remained non‑significant (Table S3). The wide confidence intervals reflect the small number of events.


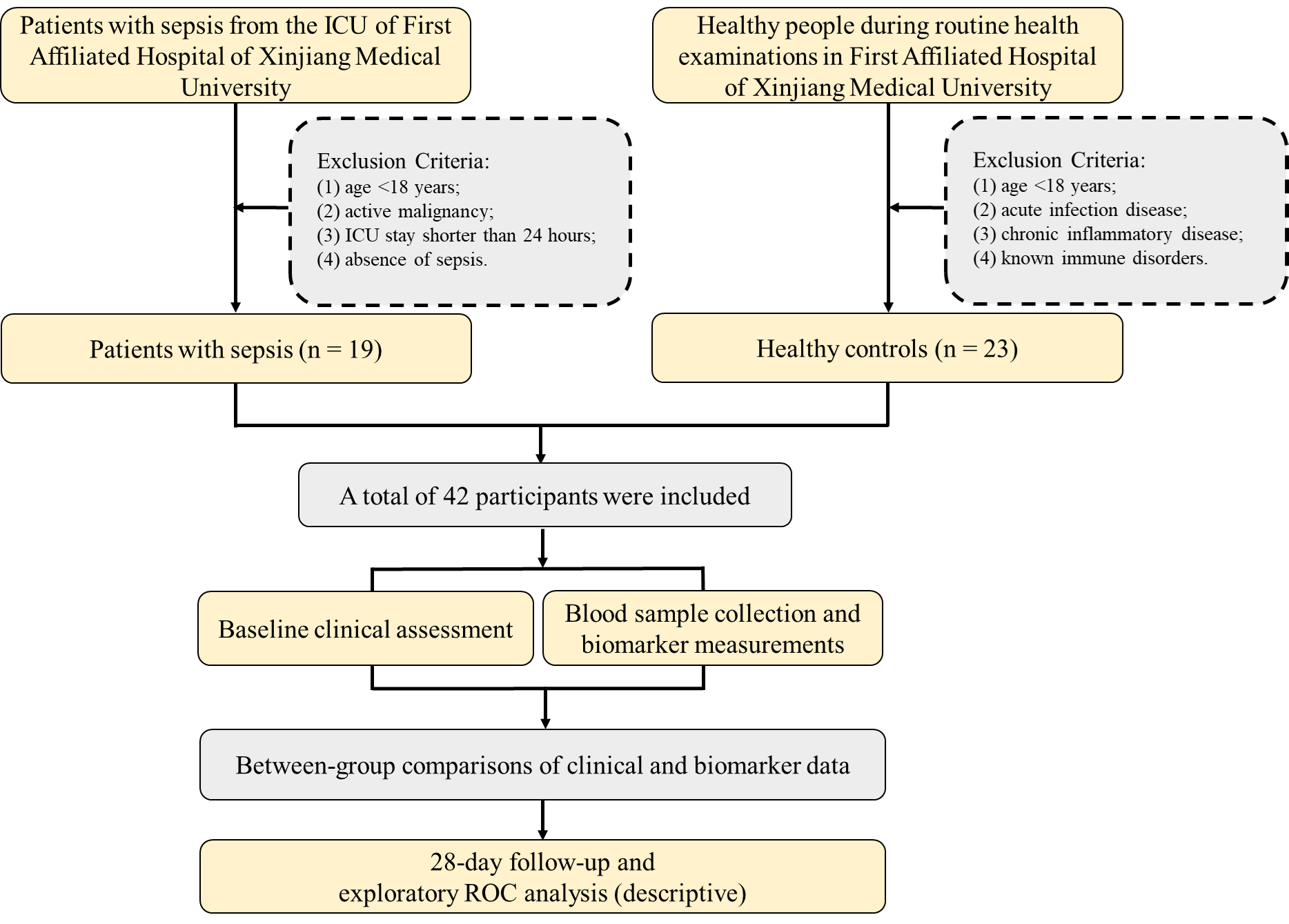


**Supplementary Figure S1** Schematic overview of patient selection and study procedures.

Adult patients admitted to the intensive care unit (ICU) of the First Affiliated Hospital of Xinjiang Medical University between September 2024 and August 2025 were screened for eligibility. Patients meeting Sepsis-3 criteria were enrolled as the sepsis cohort (n = 19) after applying predefined exclusion criteria. Healthy controls (n = 23) were recruited from individuals undergoing routine health examinations during the same period and were screened according to separate exclusion criteria. Following enrollment, a total of 42 participants were included in the final analytic cohort. Baseline clinical assessment and blood sample collection were performed at study entry. Serum biomarkers, including growth differentiation factor-15 (GDF-15), interleukin-6 (IL-6), C-reactive protein (CRP), and procalcitonin (PCT), were measured using enzyme-linked immunosorbent assays. Between-group comparisons and correlation analyses were conducted, and 28-day mortality was assessed with exploratory receiver operating characteristic (ROC) analysis.


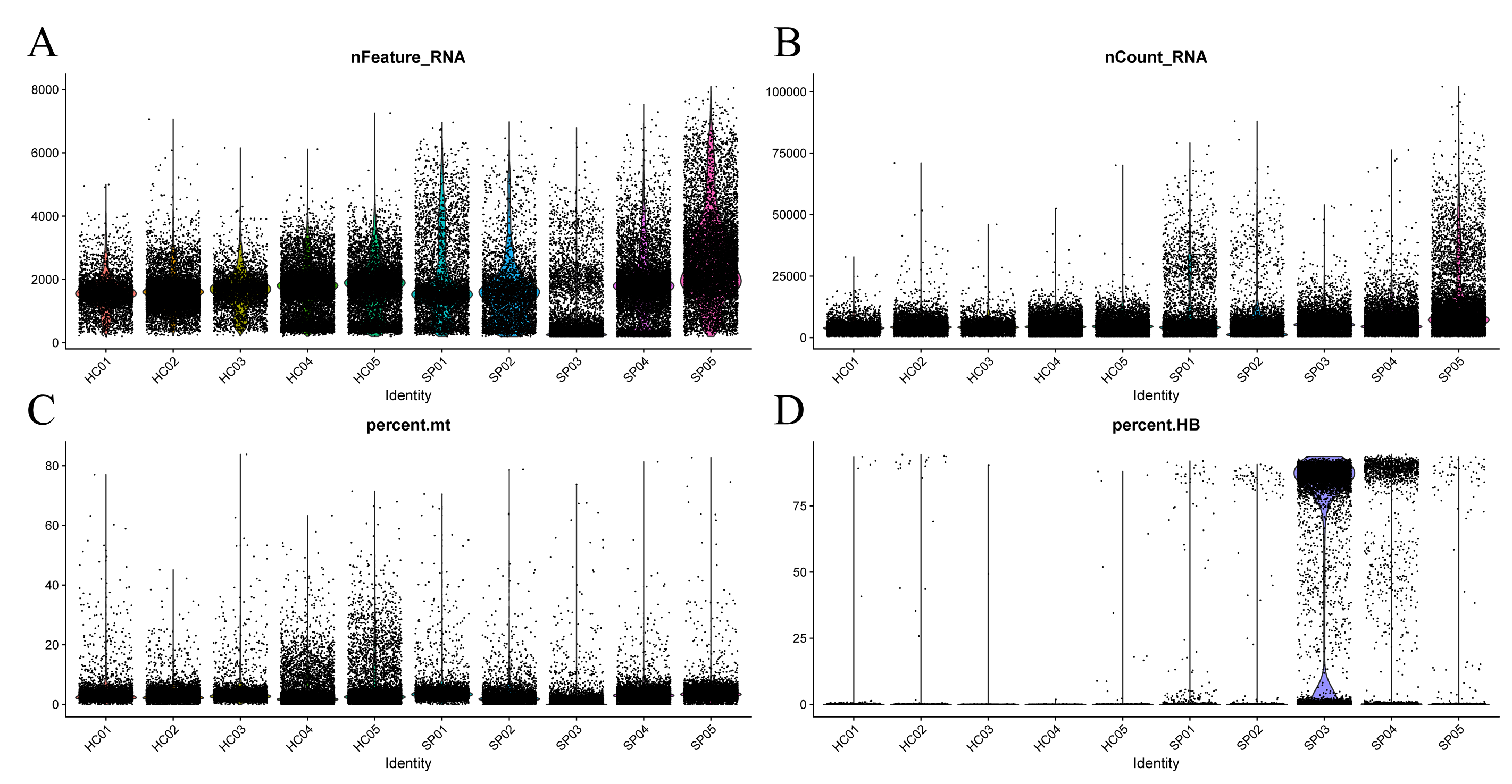


**Supplementary Figure S2. Quality control metrics of single-cell RNA sequencing data.**

Distributions of detected gene counts per cell, mitochondrial gene percentage, and hemoglobin gene percentage prior to quality filtering. Thresholds applied for downstream analyses are indicated.


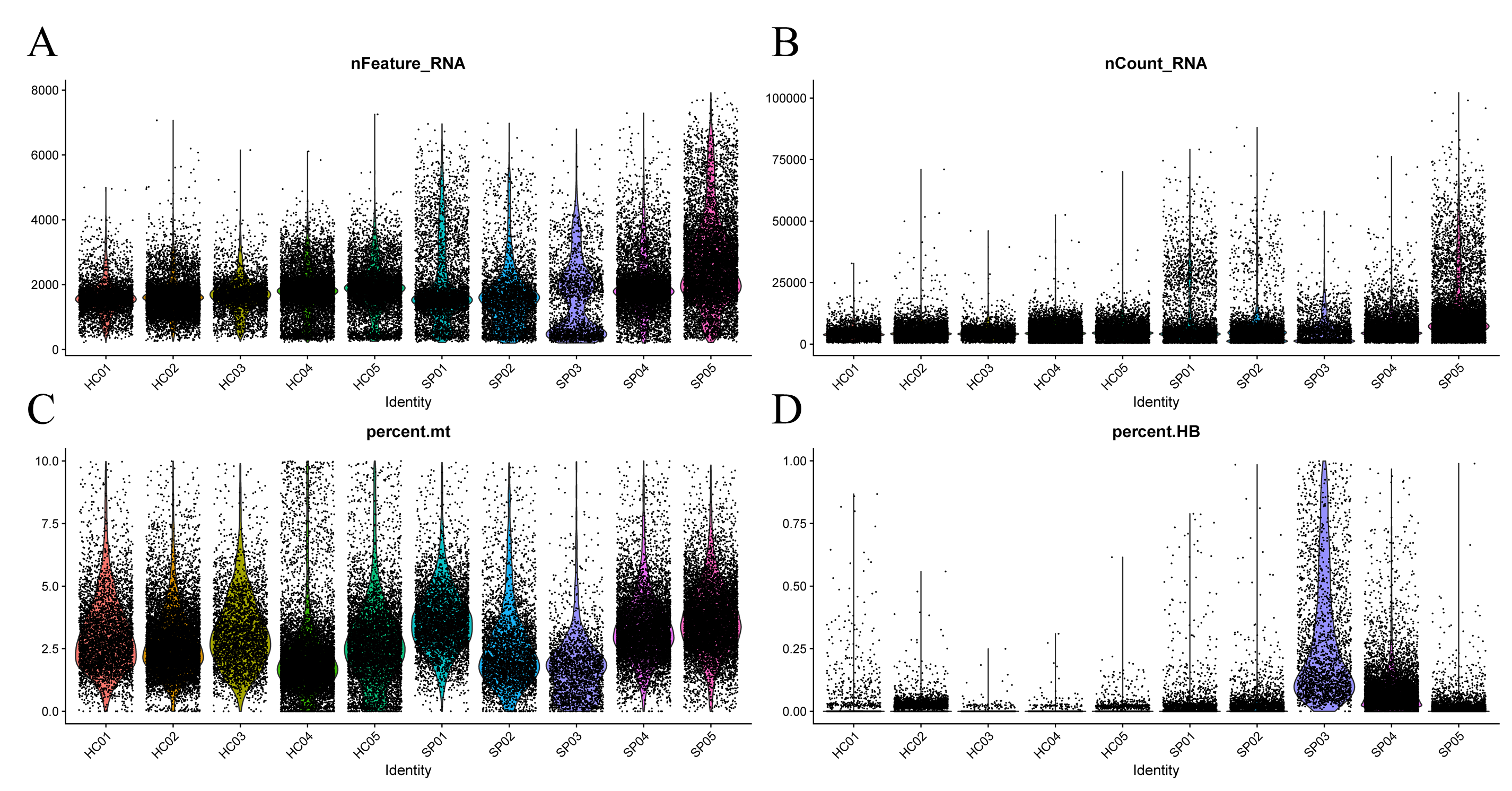


**Supplementary Figure S3. Cell filtering strategy for single-cell RNA sequencing analysis.**

Cells were excluded based on predefined quality control criteria, including low gene counts, high gene counts, elevated mitochondrial gene expression, or elevated hemoglobin gene expression, to reduce technical artifacts.


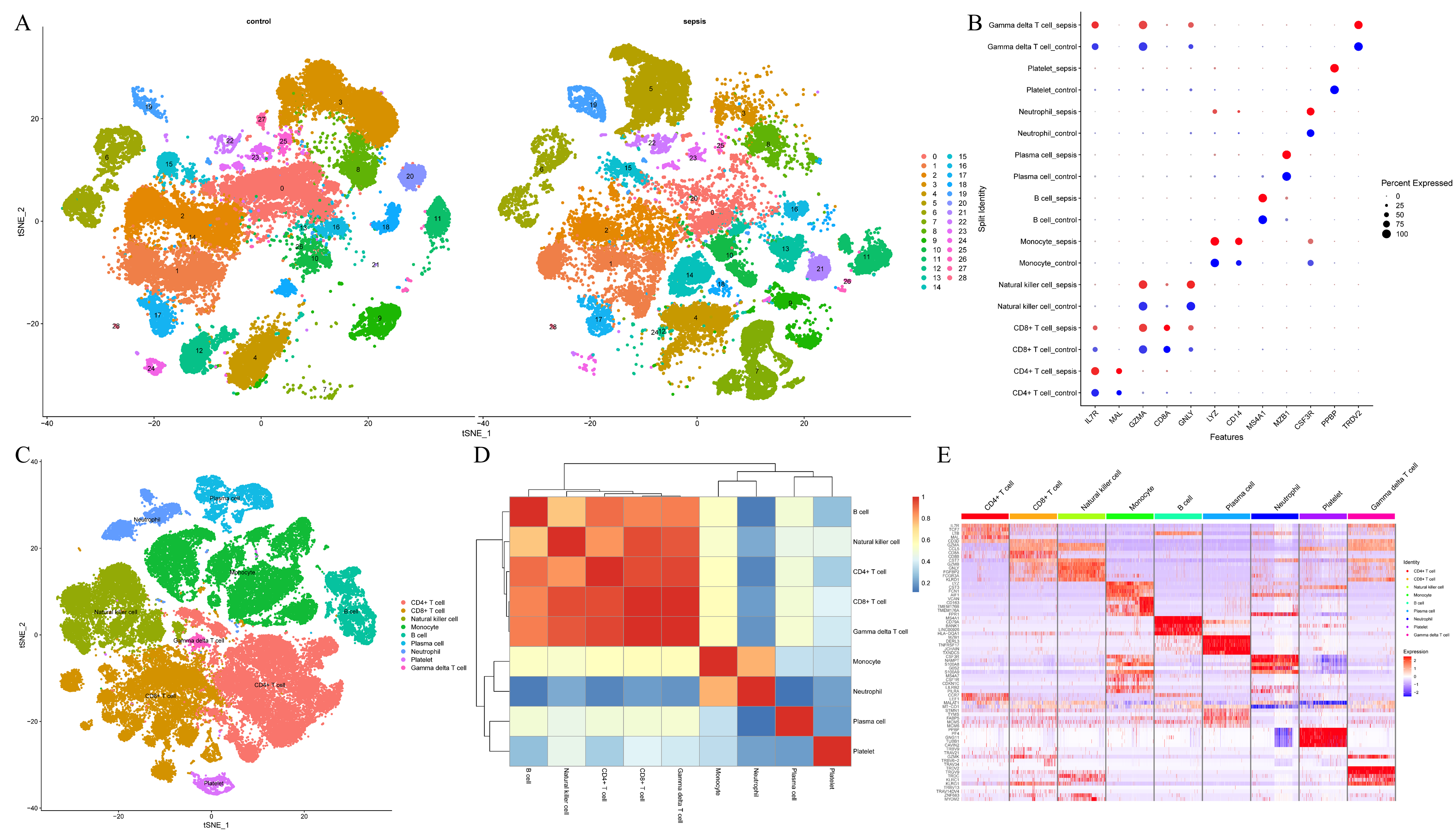


**Supplementary Figure S4. Cell-type identification and annotation of peripheral blood immune cells.**

(A–B) Feature plots and dot plots showing expression patterns of canonical marker genes used for immune cell annotation. (C) Classification of clusters into nine major immune cell populations based on established lineage-specific markers. (D) Correlation heatmap illustrating transcriptional similarity among annotated immune cell populations. (E) Heatmap displaying representative marker genes for each immune cell population. Cell type annotation was performed based on established marker gene expression and intended to provide descriptive classification of immune cell subsets.





**Supplementary Figure S5. Functional enrichment analysis of GDF15-expressing immune cell subsets.**

Gene Ontology (GO) biological process enrichment analysis of immune cell subsets expressing GDF15, performed using ClueGO. Enrichment results are presented for descriptive interpretation based on adjusted p-values following multiple testing correction.


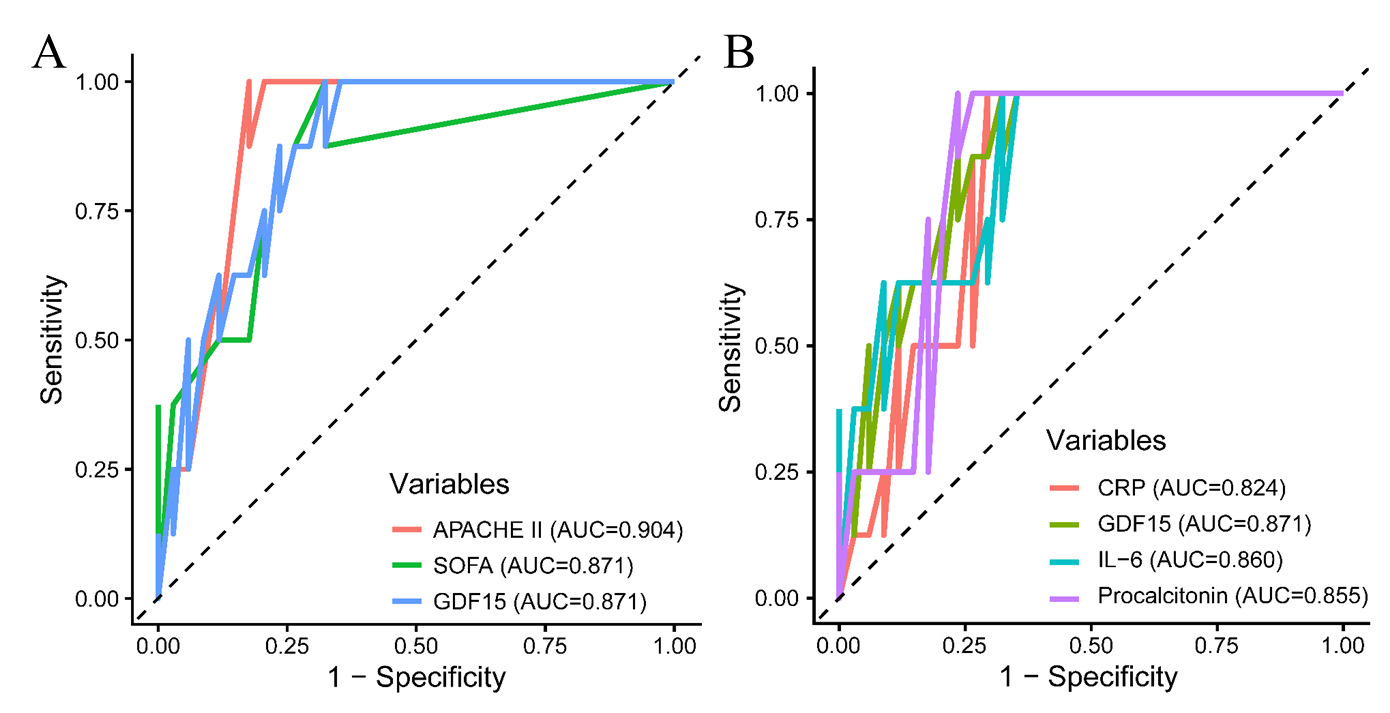


**Supplementary Figure S6. Exploratory receiver operating characteristic (ROC) analysis for 28-day mortality.**

(A) ROC curves illustrating the discriminative patterns of clinical severity scores (APACHE II and SOFA) and circulating GDF-15 levels for 28-day mortality among patients with sepsis. (B) ROC curves illustrating the discriminative patterns of inflammatory biomarkers, including C-reactive protein, interleukin-6, procalcitonin, and GDF-15. ROC analyses were conducted for exploratory visualization. Areas under the curve (AUCs) are presented with 95% confidence intervals. No cutoff optimization or multivariable modeling was performed.
